# Specific involvement of atypical PKCζ/PKMζ in spinal persistent nociceptive processing following peripheral inflammation in rat

**DOI:** 10.1186/1744-8069-7-86

**Published:** 2011-11-05

**Authors:** Fabien Marchand, Richard D'Mello, Ping K Yip, Margarita Calvo, Emilie Muller, Sophie Pezet, Anthony H Dickenson, Stephen B McMahon

**Affiliations:** 1 Neurorestoration Group, Wolfson Centre for Age-related Diseases, King's College London, Guy's Campus, London, SE1 1UL, UK; 2 Department of Neuroscience, Physiology & Pharmacology, University College London, London, WC1E 6BT, UK; 3 Laboratoire de Neurobiologie, CNRS UMR 7637, Ecole Supérieure de Physique et Chimie Industrielles, 10 rue Vauquelin, 75005 Paris, France; 4 Inserm, U 766, Clermont Université, Université d'Auvergne, Pharmacologie fondamentale et clinique de la douleur, BP 10448, F-63000 Clermont-Ferrand, France

**Keywords:** atypical PKCζ, persistent spinal nociceptive processing, inflammatory pain, dorsal horn, Fos

## Abstract

**Background:**

Central sensitization requires the activation of various intracellular signalling pathways within spinal dorsal horn neurons, leading to a lowering of activation threshold and enhanced responsiveness of these cells. Such plasticity contributes to the manifestation of chronic pain states and displays a number of features of long-term potentiation (LTP), a ubiquitous neuronal mechanism of increased synaptic strength. Here we describe the role of a novel pathway involving atypical PKCζ/PKMζ in persistent spinal nociceptive processing, previously implicated in the maintenance of late-phase LTP.

**Results:**

Using both behavioral tests and *in vivo *electrophysiology in rats, we show that inhibition of this pathway, via spinal delivery of a myristoylated protein kinase C-ζ pseudo-substrate inhibitor, reduces both pain-related behaviors and the activity of deep dorsal horn wide dynamic range neurons (WDRs) following formalin administration. In addition, Complete Freund's Adjuvant (CFA)-induced mechanical and thermal hypersensitivity was also reduced by inhibition of PKCζ/PKMζ activity. Importantly, this inhibition did not affect acute pain or locomotor behavior in normal rats and interestingly, did not inhibited mechanical allodynia and hyperalgesia in neuropathic rats. Pain-related behaviors in both inflammatory models coincided with increased phosphorylation of PKCζ/PKMζ in dorsal horn neurons, specifically PKMζ phosphorylation in formalin rats. Finally, inhibition of PKCζ/PKMζ activity decreased the expression of Fos in response to formalin and CFA in both superficial and deep laminae of the dorsal horn.

**Conclusions:**

These results suggest that PKCζ, especially PKMζ isoform, is a significant factor involved in spinal persistent nociceptive processing, specifically, the manifestation of chronic pain states following peripheral inflammation.

## Background

Peripheral nerve damage or inflammation results in the induction of molecular mechanisms within the spinal cord leading to the amplification of the pain signalling ultimately contributing to persistent pain states [1]. Long term potentiation (LTP) is a ubiquitous mechanism throughout the central nervous system underlying a long-lasting, localized increase in synaptic strength and is believed to be the neuronal substrate of learning and memory [2]. Interestingly, spinal LTP-related phenomena have also been reported in several animal pain models following either nerve damage or inflammation [3-6]. Furthermore, long-lasting enhancement of pain via high frequency stimulation in human subjects, considered to be the perceptual correlate of nociceptive LTP, have been found [7-9]. As a result, it has recently become clear that similarities and probably common intracellular signalling pathways exist between spinal persistent pain processing and LTP in the hippocampus [4,10].

PKCζ is an atypical protein kinase belonging to the protein kinase C (PKC) family, consisting of four functional domains, including regulatory domains and a kinase domain at the C-terminus [11,12]. In the brain, not only is the native form of PKCζ (75kDa) expressed, but also a smaller fragment, PKMζ (51kDa), which consists solely of the independent catalytic domain of PKCζ and is therefore persistently active. Importantly, PKMζ is, for instance, the only kinase involved in the maintenance of the late phase of LTP [12-16]. Perhaps more interestingly, during LTP in the hippocampus, PKMζ is regulated by several upstream kinases including phosphatidylinositol 3-kinase (PI3K), the mammalian target of Rapamycin (mTOR), Ca^2+^/calmodulin-dependent kinase II (CAMKII) and the extracellular signal-regulated kinase (ERK), all of which are also involved in the establishment of spinal persistent nociceptive sensitization including C-fibre-evoked spinal LTP [17-21]. Together with conventional PKC, the activation of PKCζ could contribute to the morphine-induced rewarding effect in a neuropathic pain model [22]. Moreover, along with PKCα and PKCε, PKCζ seems to be involved in sigma-1 activation induced-facilitation of nociception [23]. Finally, a very recent study investigated the role of PKMζ in a spinal sensitized state promoting pain [24]. These authors used a model consisting in an intraplantar injection of IL-6 which induced short term allodynia. The subsequent intraplantar injection of prostaglandin E2 or intrathecal injection of a glutamate receptor 1/5 agonist precipitated pain behaviors reflecting a state of persistent sensitization of the nociceptive pathway. Spinal inhibition of PKMζ at the time of intraplantar IL-6 injection or before the subsequent challenge blocked allodynia and/or nocifensive behaviors demonstrating the involvement of spinal PKMζ in the initiation and also the maintenance of a spinal sensitization state. However, the expression of both PKMζ, PKCζ and their activated form (i.e. phosphorylated) in the spinal dorsal horn, the consequence of PKMζ blockade on neuronal activity in an inflammatory pain context and its role compared to neuropathic pain have not been yet tested. Therefore, the aim of this study was to investigate the involvement of PKCζ/PKMζ in spinal persistent nociceptive processing using models of inflammatory and neuropathic pain.

## Results

### Effect of intrathecal administration of ZIP on mechanical and thermal sensitivity or locomotor function in normal rats

**Figure 1:**
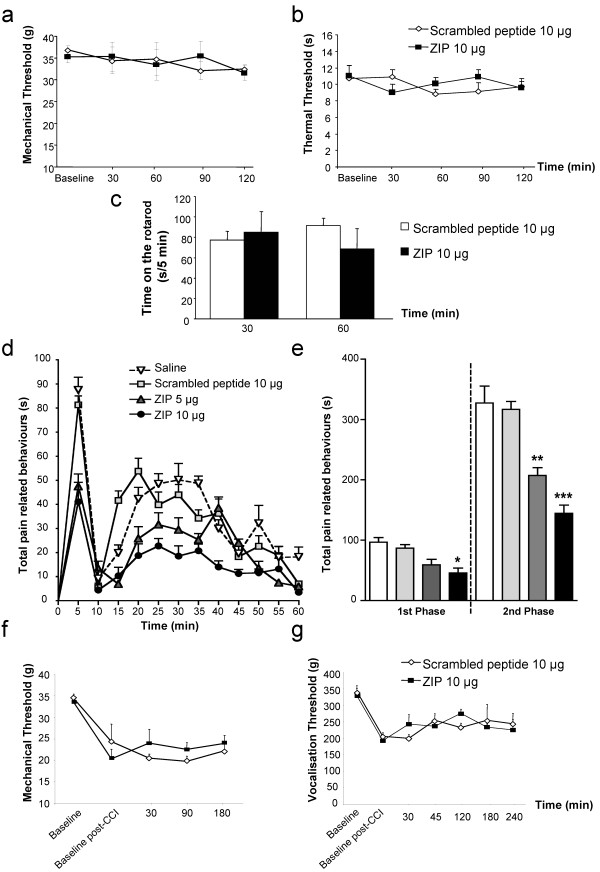
**Spinal blockade of PKCζ/PKMζ activity specifically reduces pain-related behavior induced by intraplantar formalin**. Time-course of (**a**) mechanical and (**b**) thermal sensitivity in normal rats following intrathecal administration of the scrambled peptide (10 μg, *n *= 8) and PKCζ/PKMζ pseudosubstrate inhibitor, ZIP (10 μg, *n *= 8). (**c**) Time spent on the accelerating rotarod (i.e. latency to fall) after intrathecal injection of the scrambled peptide (10 μg, *n *= 8) and ZIP (10 μg, *n *= 8). (**d**) Time-course of pain-related behaviors following subcutaneous formalin (5%, 50 μl) injection into the hindpaw following intrathecal pre-treatment with saline (*n *= 8), the scrambled peptide (10 μg, *n *= 8) or ZIP (5 μg or 10 μg, *n *= 8, ∗∗∗ *p *< 0.001, ∗∗ *p *< 0.01, ∗ *p *< 0.05 versus scrambled peptide). (**e**) Total pain-related behavior during the 1st (0-10 min) and 2nd (10-60 min) phases of the formalin response with saline, scrambled peptide or ZIP (∗∗∗ *p *< 0.001, ∗∗ *p *< 0.01, ∗ *p *< 0.05 versus scrambled peptide). Time-course of (**f**) mechanical allodynia and (**g**) mechanical hyperalgesia in CCI rats following intrathecal administration of the scrambled peptide (10 μg, *n *= 8) and ZIP (10 μg, *n *= 8). All data presented as mean ± s.e.m.

Intrathecal scrambled peptide (10 μg, *n *= 8) did not modify mechanical and thermal withdrawal responses compared to baseline in normal rats (Figure 1a, b). More interestingly, intrathecal administration of 10 μg (*n *= 8) of ZIP did not alter mechanical and thermal withdrawal responses compared to baseline and the control scrambled peptide group throughout the whole experiment (Figure 1a, b). Finally, neither the control scrambled peptide nor ZIP had any effect on the locomotor function of rats on the rotarod, assessed at 30 and 60 min post-injection (Figure 1c). We subsequently examined if spinal PKCζ was involved in pain following peripheral inflammation or nerve injury.

### Effect of intrathecal administration of ZIP on pain-related behaviors induced by intraplantar formalin

In control rats pre-treated spinally with either saline (*n *= 8) or the scrambled peptide (10 μg, *n *= 8), formalin injection induced a similar biphasic response of pain-related behaviors, as expected (Figure 1d,e; saline: 1^st ^phase (0-10 min), = 97 ± 8 s; 2^nd ^phase (10-60 min) = 328 ± 28 s; scrambled peptide: 1^st ^phase = 87 ± 6 s; 2^nd ^phase = 317 ± 13 s). In contrast, pre-treatment with ZIP, especially at the highest dose, significantly decreased pain-related behaviors during both the first and second phases of the response to formalin (Figure 1d,e; 5 μg: *n *= 6, 1^st ^phase = 59 ± 9 s, *p *> 0.05; 2^nd ^phase = 207 ± 13 s, *p *< 0.001; 10 μg: *n *= 9, 1^st ^phase = 46 ± 8 s, *p *< 0.01; 2^nd ^phase = 145 ± 14 s, *p *< 0.001) compared to the scrambled peptide group. As the high dose (10 μg) of ZIP exhibited a clear antinociceptive effect in this inflammatory pain model, this dose was tested in a model of neuropathic pain, the chronic constriction injury (CCI).

### Effect of intrathecal administration of ZIP on mechanical allodynia and hyperalgesia in CCI rats

Chronic constriction injury of the sciatic nerve significantly decreased mechanical withdrawal responses and vocalisation thresholds as expected (Figure 1f,g). Intrathecal scrambled peptide (10 μg, *n *= 8) did not modify mechanical withdrawal responses and vocalisation thresholds compared to baseline post-CCI in CCI rats (Figure 1f,g). More interestingly, intrathecal administration of 10 μg (*n *= 8) of ZIP did not significantly inhibit mechanical withdrawal responses and vocalisation thresholds compared to baseline post-CCI and also the control scrambled peptide group throughout the whole experiment, indicating a lack of effect of ZIP on both mechanical allodynia and hyperalgesia following nerve injury.

Thus, PKCζ/PKMζ seems to be specifically involved in inflammatory pain and not in acute pain and, interestingly, neuropathic pain. Following these behavioral results, we have studied the effect of spinal application of ZIP on deep dorsal horn neurons activity using electrophysiology following formalin administration, and localisation, expression of PKCζ/PKMζ.

### Effect of spinal application of ZIP on the formalin-induced firing response of deep dorsal horn WDR neurons

**Figure 2:**
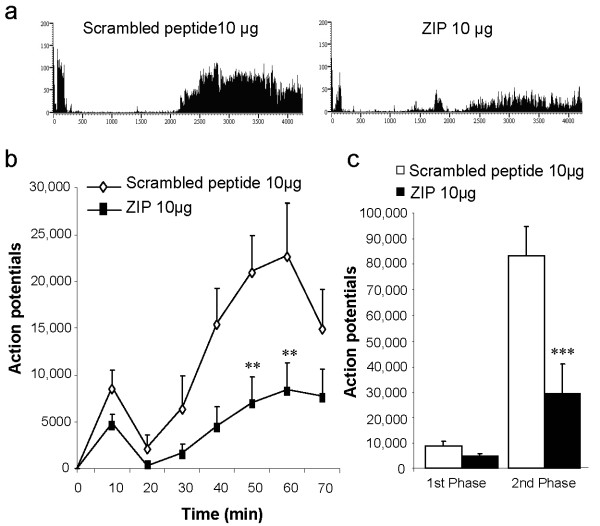
**Spinal blockade of PKCζ/PKMζ activity reduces the response of deep dorsal horn WDR neurons induced by intraplantar formalin**. (**a**) Representative rate recordings of firing responses of WDR neurons to formalin following spinal pre-treatment with scrambled peptide or ZIP. (**b**) Time-course of WDR firing response to subcutaneous formalin (5%, 50 μl) injection into the hindpaw receptive field following spinal pre-treatment with scrambled peptide (10 μg, *n *= 8) or ZIP (10 μg, *n *= 8, ∗∗ *p *< 0.01 versus scrambled peptide). (**c**) Total neuronal activity during the 1st (0-10 min) and 2nd (10-70 min) phases of the formalin response with scrambled peptide or ZIP (2^nd ^phase: ∗∗∗ *p *< 0.001 versus scrambled peptide). Results are expressed as mean ± s.e.m.

In control rats pre-treated with the scrambled peptide (10 μg, n = 8), formalin injected into the hindpaw receptive field produced the characteristic biphasic neuronal firing response of spinal WDR neurons (Figure 2a-c; 1st phase: 0-10 min, total APs = 8743 ± 1778; 2^nd ^phase: 10-70 min, total APs = 83358 ± 11531). Spinal pre-treatment with ZIP (10 μg, *n *= 8) reduced both first and second phase neuronal firing, though this effect was only statistically significant on second phase activity (Figure 2a-c; 1st phase: total APs = 4847 ± 979, *p *> 0.05; 2^nd ^phase: total APs = 29643 ± 11132, *p *< 0.001).

### Expression and localisation of PKCζ and phospho-PKCζ/p- PKMζ in the spinal cord

**Figure 3:**
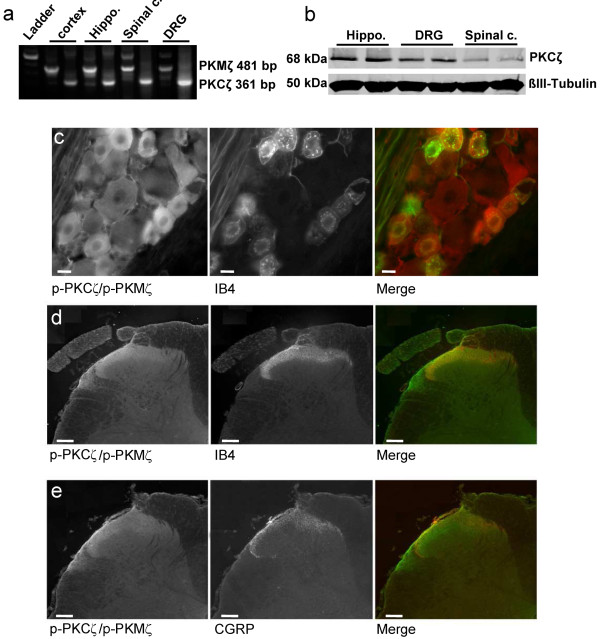
**Expression of PKCζ/PKMζ and phospho-PKCζ/PKMζ**. (**a**) RT-PCR showing expression of PKMζ (481 bp) and PKCζ (361 bp) mRNA isoforms in spinal cord (spinal c.), dorsal roots ganglia (DRG) and in both cortex and hippocampus (Hippo.) in naïve rats. (**b**) Western immunoblot showing expression of PKCζ in hippocampus (Hippo.), DRG and lumbar spinal dorsal horn (Spinal c.) in naïve rats. ß-III tubulin served as loading control. (**c**) p-PKCζ/p-PKMζ is co-expressed with IB4 in DRG cells of naïve rats. Scale bars = 50 μm. (**d, e**) p-PKCζ/p-PKMζ is not co-expressed with (**d**) IB4 or (**e**) CGRP, used as specific markers for non-peptidergic and peptidergic C-fibers, respectively, in spinal cord sections from formalin rats. Scale bars = 100 μm.

To our knowledge no specific antibody only against PKMζ is commercially available. Therefore, we used RT-PCR to identify which mRNA isoforms of PKCζ are expressed in the DRG and spinal cord of naïve rats. We found 2 bands at 481 bp and 361 bp as expected for PKMζ and PKCζ mRNA isoforms, respectively, in our spinal cord and DRG samples but also in our positive control samples (i.e. cortex, hippocampus) suggesting that both mRNA isoforms, and probably proteins, are expressed in the DRG and spinal cord (Figure 3a).

**Figure 4:**
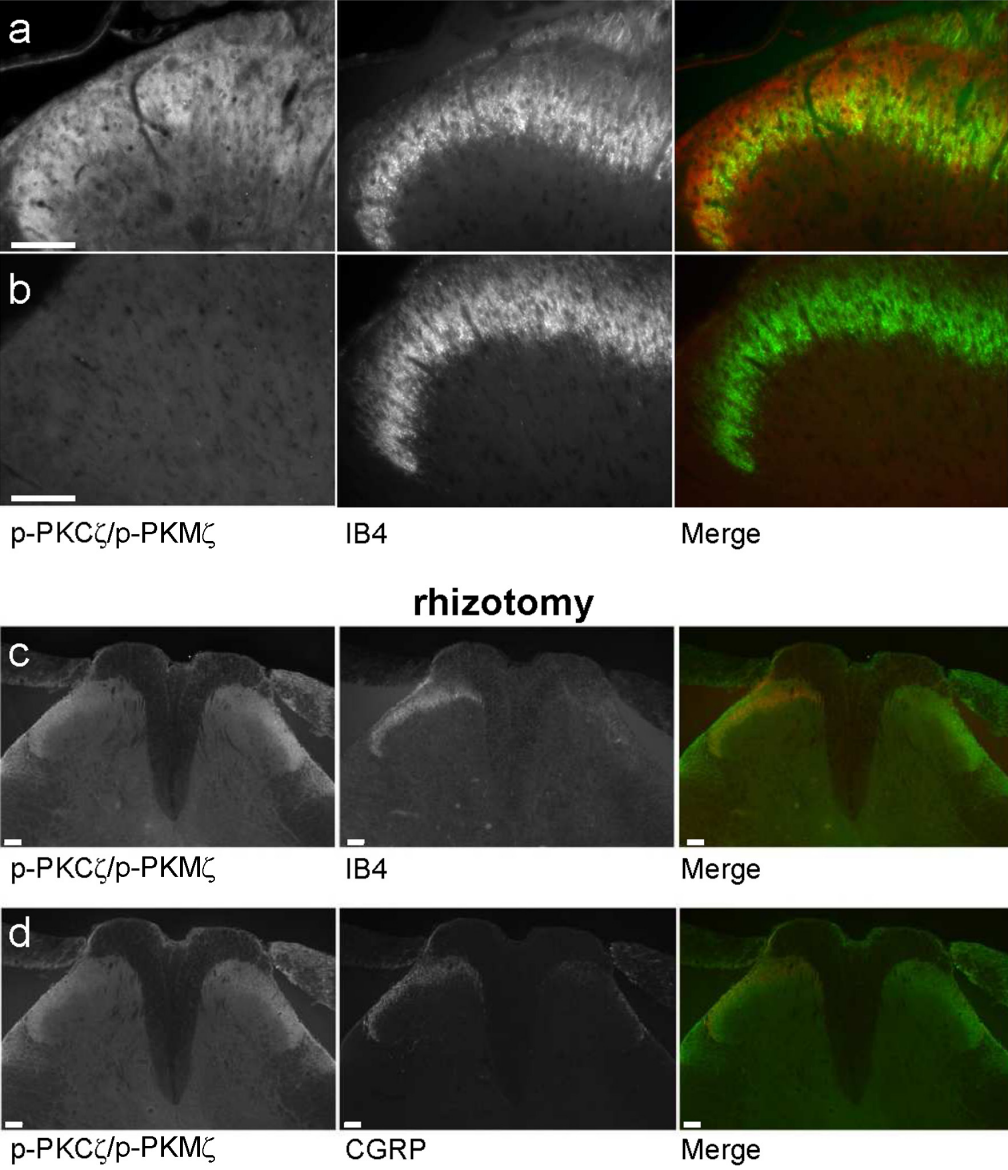
**Expression of phospho-PKCζ/PKMζ following lumbar rhizotomy**. High power magnification of p-PKCζ/p-PKMζ expression with IB4 in spinal cord sections from formalin rats (**a**). (**b**) The zeta-specificity of the antibody used is shown using the blocking peptide which drastically diminished p-PKCζ/p-PKMζ staining (left panel). (**c**, **d**) Lumbar rhizotomy caused an almost total loss of (**c**) IB4 and (**d**) CGRP staining demonstrating substantial deafferentation. However, p-PKCζ/p-PKMζ staining still remained. Scale bars = 100 μm.

By western immunoblotting, we investigated the expression of PKCζ and PKMζ in the DRG, spinal dorsal horn and hippocampus using a specific anti-PKCζ antibody (Figure 3b). Western blots analysis confirmed that PKCζ protein is detected in the hippocampus, DRG and importantly, spinal dorsal horn (Figure 3b). Using an antibody against PKCζ/PKMζ active form, i.e. phospho-PKCζ/p-PKMζ, for which we have characterized the specificity using the blocking peptide (Figure 4b), we observed that p-PKCζ/p-PKMζ are expressed in all categories of DRG neurons, with a highest intensity of immunostaining in the cytoplasm and membrane of small and medium DRG neurons of naïve rats (Figure 3c). We then investigated if p-PKCζ/p-PKMζ is transported to and/or expressed in primary afferent central terminals within the dorsal horn in formalin animals. Expression of p-PKCζ/p-PKMζ was observed in the superficial dorsal horn but we did not observe any obvious co-localisation of p-PKCζ/p-PKMζ with IB4 and CGRP, specific markers for non-peptidergic and peptidergic C-fibres, respectively (Figure 3d,e), even at high power magnification (Figure 4a). In addition, we used lumbar rhizotomy surgery in a separate experiment to see if p-PKCζ/p-PKMζ are expressed in primary afferent fibre terminals as we observed an almost total loss of IB4 and CGRP staining following rhizotomy, illustrating substantial deafferentation (Figure 4c,d). Interestingly, we did not observe any change in spinal p-PKCζ/p-PKMζ staining, indicating a lack of expression of p-PKCζ/p-PKMζ in primary afferent fibres terminals (Figure 4c,d) and suggesting a predominantly intrinsic spinal source.

**Figure 5:**
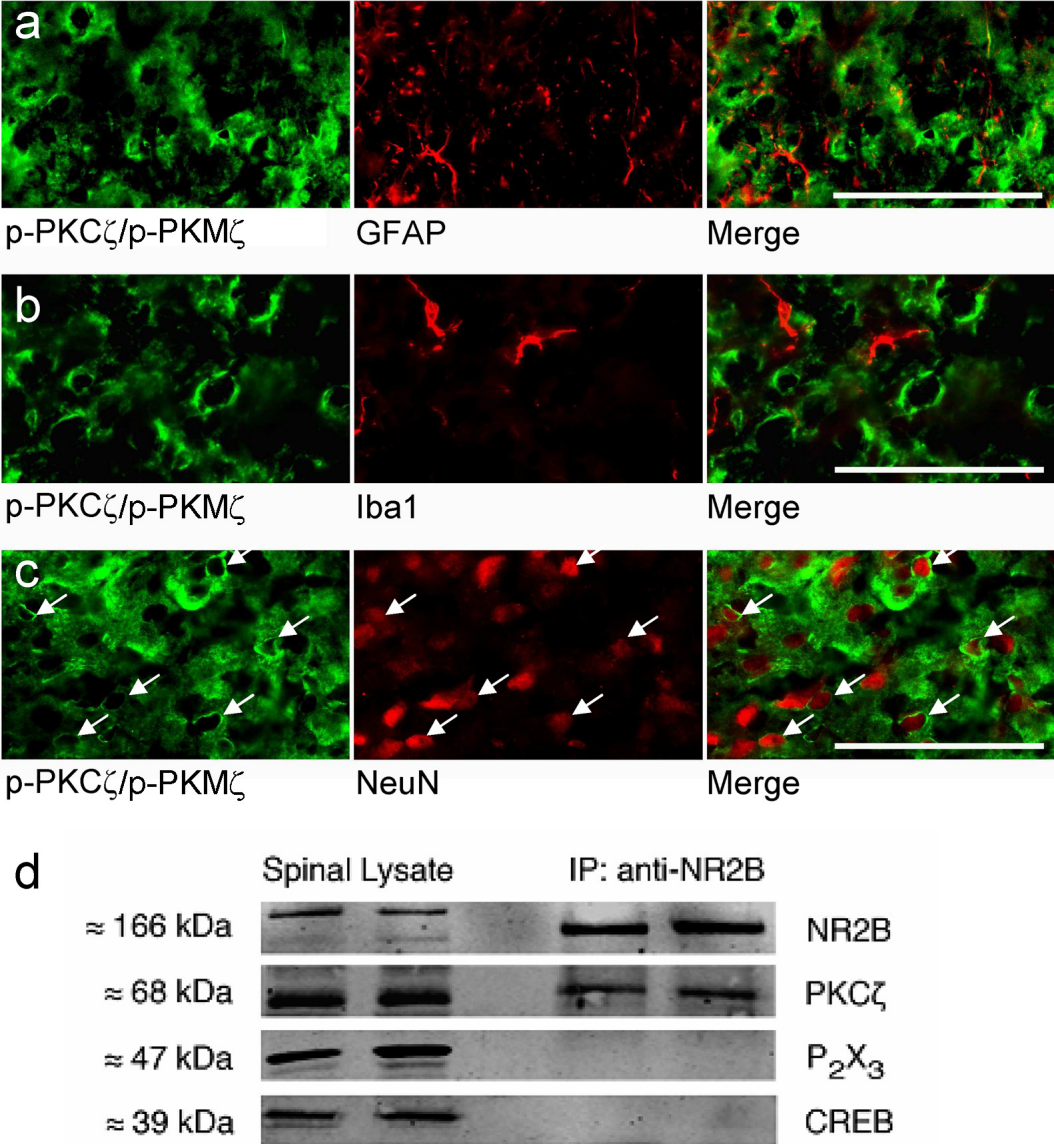
**Phospho-PKCζ/PKMζ is expressed in spinal dorsal horn neurons**. (**a-c**) p-PKCζ/p-PKMζ did not co-localize with (**a**) GFAP or (**b**) Ib1a but did co-localize with (**c**) NeuN. Scale bars = 50 μm. (**d**) Western immunoblots of naïve rats' lumbar dorsal horn lysates (left two lanes) and co-immunoprecipitates from lumbar dorsal horn lysates obtained using an antibody against NR2B (right two lanes), probed with antibodies against NR2B, PKCζ, CREB and P2X3.

Consequently, we attempted to identify the phenotype of p-PKCζ/p-PKMζ-expressing cells in spinal cord sections from formalin rats (Figure 5a-c). Phospho-PKCζ/p-PKMζ immunoreactivity was observed in NeuN-positive cells, marking neuronal nuclei (Figure 5c). No obvious co-localization was observed with GFAP-positive astrocytes or Iba1-positive microglial cells (Figure 5a, b). In addition, we used immunoprecipitation to reveal a physical coupling between PKCζ and NR2B subunits, which are specifically expressed in intrinsic dorsal horn sensory neurons and are also essential for spinal nociceptive plasticity (Figure 5d). PKCζ protein was co-immunoprecipitated by the anti-NR2B antibody. Normal spinal lysate was run alongside immunoprecipitation samples as positive controls, while CREB and P2X3 were used as two negative control proteins, to verify that the immunoprecipitation step had worked. The P2X3 receptor is located exclusively on the central terminals of primary afferents and CREB is a nuclear protein, therefore these two proteins should not be found in a post-synaptic, membrane-associated NR2B/PKCζ complex (Figure 5d). Indeed, both P2X3 and CREB were only found in normal spinal tissue lysates, confirming that non-specific detection of proteins in the immunoprecipitation sample had not occurred. Thus, PKCζ is physically coupled to NR2B-containing NMDA receptors in the spinal dorsal horn.

Together, these results suggest that phosphorylation of PKCζ/PKMζ following intraplantar formalin is mainly within intrinsic spinal dorsal horn neurons and could occurred in NR2B containing NMDA receptors neurons. However, phosphorylation of PKCζ/PKMζ needed to be quantified.

### Effect of intraplantar formalin on the phosphorylation of PKCζ/PKMζ expression in the spinal cord

**Figure 6:**
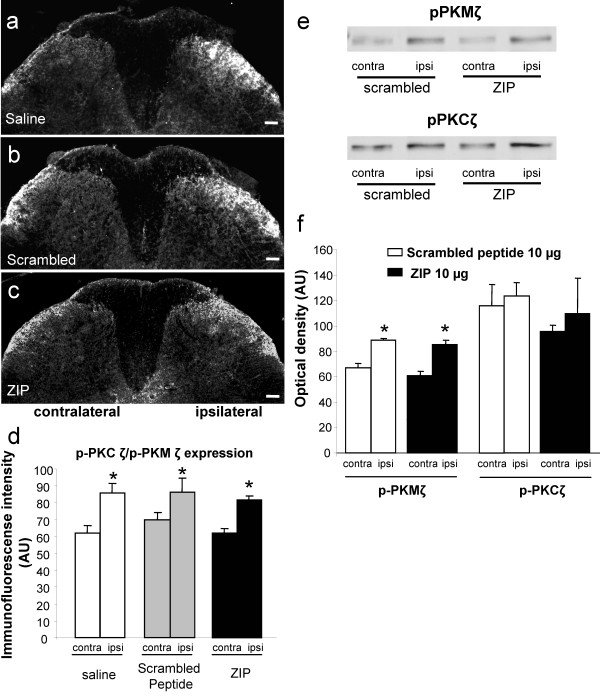
**Effect of spinal PKCζ/PKMζ inhibition on PKCζ/PKMζ phosphorylation in the dorsal horn following intraplantar formalin**. (**a**-**c**) Representative photomicrographs of increased expression of p-PKCζ/p-PKMζ in superficial laminae of the ipsilateral lumbar dorsal horn following intraplantar formalin in rats pre-treated with intrathecal (**a**) saline, (**b**) scrambled peptide (10 μg, *n *= 5) or (**c**) ZIP (10 μg, *n *= 5). Scale bars = 100 μm. (**d**) Quantification of p-PKCζ/p-PKMζ expression in lumbar dorsal horn presented as mean ± s.e.m. of immunofluorescent intensity (arbitrary unit) of p-PKCζ/p-PKMζ staining in ipsilateral and contralateral lumbar dorsal horn for each group. (**e, f**) Western immunoblots showing phosphorylation of PKCζ and PKMζ in formalin rats. Intraplantar injection of formalin induced a significant increase of ipisilateral PKMζ phosphorylation (e top panel, f) but not PKCζ (e bottom panel, f) compared with the contralateral side in the scrambled peptide treated group. Intrathecal injection of ZIP (10 μg) did not change the increased phosphorylation of PKMζ (e top panel, f). Results are expressed as mean ± s.e.m. of the densitometric analysis (arbitrary unit) of p-PKCζ and p-PKMζ expression levels in the ipsilateral and contralateral lumbar dorsal horn for each group.

Intraplantar formalin injection in the saline (10 μl, *n *= 5) and scrambled peptide groups (10 μg, *n *= 5) produced a significant increase of p-PKCζ/p-PKMζ in the superficial (laminae I-II) layers of the ipsilateral dorsal horn (levels L4-L5) compared to the contralateral side (mean percentage increase = 27% ± 3.4 and 22% ± 2, respectively, *p *< 0.05; Figure 6a,b,d). In addition, intrathecal injection of ZIP (10 μg, *n *= 5) did not significantly alter formalin-induced PKCζ/PKMζ phosphorylation (mean percentage increase = 25% ± 2; Figure 6c,d).

In addition to immunohistochemistry quantification, we performed western blot analysis to assess the effect of intraplatar formalin and also PKCζ/PKMζ inhibition on the phosphorylation of PKCζ and PKMζ in L4-L5 spinal dorsal horn, 60 min after formalin injection. Scrambled-treated rats exhibited a significant increase in p-PKMζ but not p-PKCζ expression compared with the contralateral dorsal horn (*p *< 0.05; Figure 6e,f). Intrathecal administration of ZIP did not alter the phosphorylation of PKMζ compared with scrambled-treated rats (Figure 6e,f). As p-PKCζ expression did not significantly increase in the scrambled group following formalin, ZIP was without any effect (Figure 6e,f).

### Effect of intraplantar formalin on Fos expression in the spinal cord

**Figure 7:**
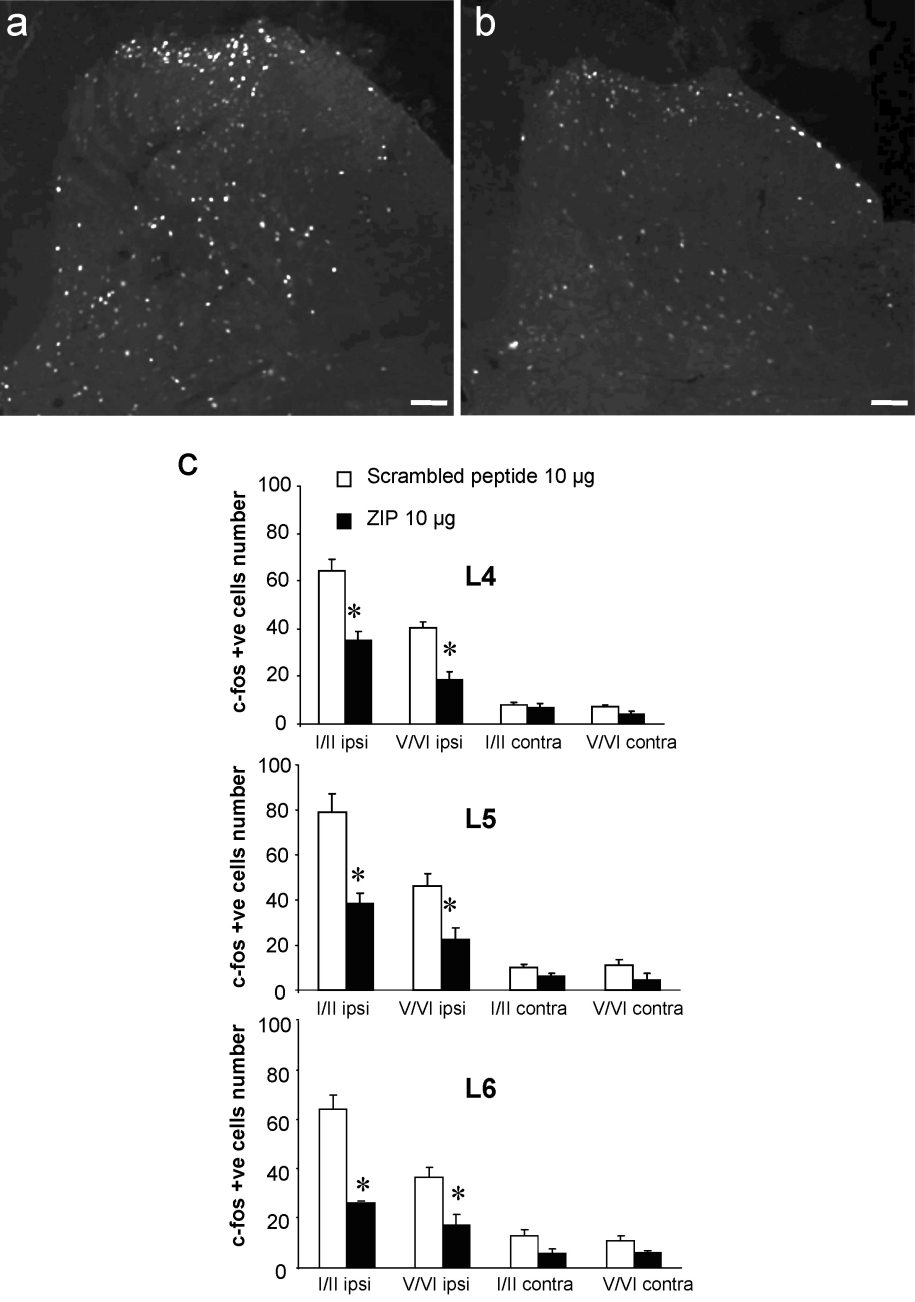
**Effect of spinal PKCζ/PKMζ inhibition on Fos expression in the dorsal horn following intraplantar formalin**. (**a, b**) Representative photomicrographs of formalin-induced upregulation of Fos expression in superficial (I-II) and deep laminae (V-VI) of the ipsilateral lumbar dorsal horn in rats pre-treated with intrathecal (**a**) scrambled peptide (10 μg, *n *= 5) or (**b**) ZIP (10 μg, *n *= 5). (**c**) Quantification of number of Fos-positive cells in superficial and deep laminae of the lumbar (L4-L6) dorsal horn. All data presented as mean ± s.e.m., ∗ *p *< 0.05. Scale bars = 100 μm.

Finally, we evaluated the effect of ZIP on Fos expression which marks activated neurons. The number of Fos positive cells in superficial (I-II) and deep (V-VI) laminae of the lumbar dorsal horn (L4, L5 and L6) was increased at 60 min after intraplantar formalin injection in scrambled peptide-treated rats (10 μg, *n *= 5; Figure 7a,c). In contrast, intrathecal pre-treatment with ZIP (10 μg, *n *= 5) resulted in a significant reduction of Fos-positive cells in both the superficial (L4: 45.5% ± 5.8 reduction; L5: 51.3% ± 5.9 reduction; L6: 59.3% ± 1.1 reduction, all *p *< 0.001) and deep dorsal horn (L4: 53.2% ± 8.4 reduction; L5: 51.6% ± 9.2 reduction; L6: 52.9% ± 11.4 reduction, all *p *< 0.001) compared to the scrambled peptide group (Figure 7b,c).

### Effect of intrathecal administration of ZIP on mechanical and thermal hypersensitivity induced by intraplantar CFA

**Figure 8:**
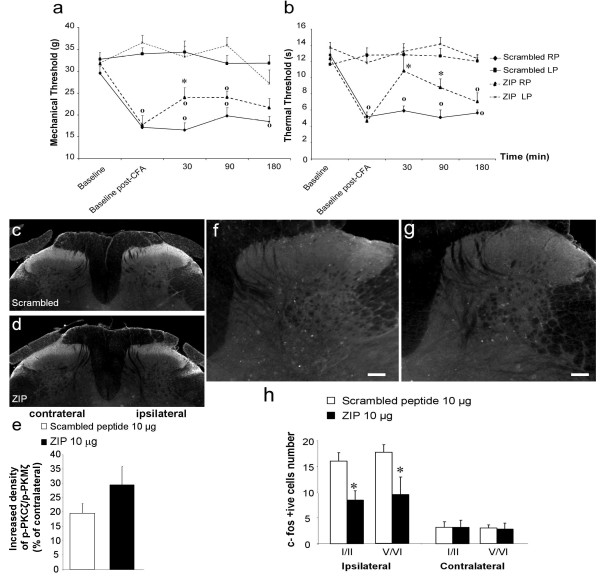
**Effect of spinal PKCζ/PKMζ inhibition on pain-related behaviors, PKCζ/PKMζ phosphorylation and Fos expression in spinal neurons following intraplantar CFA**. Time-course of (**a**) mechanical and (**b**) thermal hypersensitivity in CFA rats following intrathecal administration of the scrambled peptide (10 μg, *n *= 8) or ZIP (10 μg, *n *= 8). Results are expressed as mean ± s.e.m. (∗ *p *< 0.05 versus scrambled peptide and **o ***p *< 0.05 versus the contralateral paw). (**c**, **d**) Representative photomicrographs of increased expression of p-PKCζ/p-PKMζ in superficial laminae of the ipsilateral lumbar dorsal horn following intraplantar CFA in rats pre-treated with intrathecal (**c**) scrambled peptide (10 μg, *n *= 5) or (**d**) ZIP (10 μg, *n *= 5). (**e**) Quantification of increased expression of p-PKCζ/p-PKMζ in the ipsilateral dorsal horn presented as a percentage of contralateral dorsal horn expression. (**f**, **g**) Representative photomicrographs of CFA-induced upregulation of Fos expression in superficial (I-II) and deep laminae (V-VI) of the ipsilateral lumbar dorsal horn in rats pre-treated with intrathecal (**f**) scrambled peptide (10 μg, *n *= 5) or (**g**) ZIP (10 μg, *n *= 5). (**h**) Quantification of number of Fos-positive cells in superficial and deep laminae of the lumbar (L4-L6) dorsal horn. All data presented as mean ± s.e.m. ∗ *p *< 0.05. Scale bars = 100 μm.

Next, we used a more sustained model of inflammation-induced spinal nociceptive plasticity, produced by intraplantar injection of CFA. Twenty-four hours after CFA administration, we observed a significant decrease of mechanical withdrawal thresholds before treatment in all groups (Figure 8a). Intrathecal scrambled peptide administration (10 μg, *n *= 8) did not modify mechanical withdrawal thresholds, which remained significantly different from baseline throughout the whole experiment. In contrast, intrathecal administration of 10 μg of ZIP (*n *= 8) significantly increased mechanical withdrawal thresholds 30 min after its administration in comparison to the scrambled peptide-treated group (increase of 41% ± 14, *p *< 0.05; Figure 8a). However, mechanical withdrawal thresholds of the ipsilateral paw still differed significantly from that of the contralateral paw (*p *< 0.05; Figure 8a). Finally, ZIP had no effect on mechanical withdrawal thresholds at 90 and 180 min post-injection.

CFA administration also reduced thermal withdrawal latencies before treatment in all groups (Figure 8b). Intrathecal scrambled peptide administration did not alter thermal withdrawal latencies from the baseline throughout the test period (Figure 8b). In contrast, intrathecal administration of 10 μg of ZIP significantly increased thermal withdrawal latencies at 30 and 90 min post-injection compared to the scrambled peptide group (30 min: increase of 82.7% ± 31, *p *< 0.001; 90 min: increase of 73.5% ± 21, *p *< 0.05; Figure 8b). Thermal withdrawal latencies of the ipsilateral paw of ZIP-treated group still remained significantly different from those of the contralateral paw at 90 and 180 min.

### Effect of intraplantar CFA on the phosphorylation of PKCζ/PKMζ and Fos expression in the spinal cord

Intraplantar CFA injection in the scrambled peptide group (10 μg, *n *= 5) produced a significant increase of p-PKCζ/p-PKMζ in the superficial (laminae I-II) layers of the ipsilateral dorsal horn (levels L4-L5) compared to the contralateral side (mean percentage increase = 19.6% ± 3.3, *p *< 0.05; Figure 8c,e). As in the formalin experiment, intrathecal injection of ZIP (10 μg, *n *= 5) did not reduce CFA-induced PKCζ/PKMζ phosphorylation (mean percentage increase = 29.4% ± 6.4; Figure 8d,e).

We also examined the effect of intrathecal administration of ZIP (10 μg, *n *= 5) on CFA-induced upregulation of Fos expression. In CFA animals treated with the scrambled peptide, we observed a significant increased expression of Fos in superficial (I-II) and deep (V-VI) laminae of the ipsilateral lumbar dorsal horn compared to the contralateral side (Figure 8f,h). In contrast, spinal delivery of ZIP significantly reduced CFA-induced Fos expression in superficial (47.2% ± 11.2 reduction) and deep (46.5% ± 19.4 reduction) laminae of the ipsilateral lumbar dorsal compared to the control scrambled peptide-treated group (Figure 8g,h).

## Discussion

This study demonstrates that atypical PKCζ more specifically the PKMζ isoform is involved in spinal persistent nociceptive processing only following peripheral inflammation. Indeed, a specific pseudosubstrate inhibitor of PKCζ/PKMζ, ZIP, injected intrathecally, reduced pain-related behaviors elicited by intraplantar injection of formalin and CFA while it did not modify mechanical and thermal sensitivity or locomotor function in normal rats and interestingly, mechanical allodynia and hyperalgesia in a neuropathic pain model. Furthermore, direct spinal application of this inhibitor reduced the firing response of WDR neurons to formalin administration into the hindpaw receptive field during the second phase. Pain behaviors in both inflammatory models were associated with increased expression of the activated form (i.e. phosphorylated) of PKCζ, especially phospho-PKMζ in the ipsilateral dorsal horn of formalin rats, suggested to be specifically within spinal neurons. Finally, inhibition of PKCζ/PKMζ decreased Fos expression induced by peripheral inflammation in both superficial and deep laminae of the lumbar spinal dorsal horn.

Numerous studies have shown increased translocation and expression of different forms of PKC in dorsal horn neurons in pain models (for review see [25]). Moreover, inhibition of PKCs using non-specific PKC inhibitors (e.g. chelerythrine) reduces inflammation-induced pain related behaviors [26-28]. PKCε may have a role in neurotransmitter release from primary afferent terminals [29], while PKCγ only contributes to increased hyperexcitability of a subset of lamina V NMDA-dependent neurons following an inflammatory stimulus, suggesting only a partial contribution of this kinase in spinal sensitized state, especially in mediating mechanical hypersensitivity [30-32]. Therefore, other PKCs must be involved, with PKMζ a likely contributor since it is involved in the maintenance of hippocampal LTP [13,14]. Thus, we sought to investigate the function of PKCζ/PKMζ in naïve animals and in both inflammatory and neuropathic pain models.

Mechanical and thermal nociceptive thresholds as well as locomotor function in normal rats were unaffected by spinal PKCζ/PKMζ inhibition by ZIP as also shown for acute mechanical sensitivity [23,24]. However, pain-related behaviors in the first and second phases of the formalin test were significantly reduced following intrathecal administration of ZIP. This effect observed on the first phase might suggest that PKCζ/PKMζ is involved in acute pain but, as demonstrated, ZIP did not affect nociception in normal rats. The biphasic behavioral response seen in freely moving animals is mimicked by the biphasic firing response of WDR neurons to peripheral formalin [26,33]. Using this electrophysiological approach, we observed that the second phase of the formalin test considered to underlie spinal nociceptive sensitization was substantially reduced by spinal ZIP application, indicating an inhibitory effect on neuronal excitability. In contrast, no significant effects were seen during the first phase of neuronal response. Monitoring both the behavioral and neuronal measures, there is a remarkable concordance, yet the second phase of the formalin response was reduced by ZIP but only the first phase in awake animals. There was a clear but insignificant reduction in first phase neuronal firing which may be enough to result in reduced withdrawal responses in awake animals. We subsequently utilized a more sustained model of inflammatory pain induced by intraplantar CFA, which exhibited significant reduction of their nociceptive thresholds within 24 hours, indicating the establishment of spinal persistent nociceptive sensitization. Intrathecal administration of ZIP significantly inhibited both mechanical and thermal hypersensitivity. In contrast to the formalin test, there are few studies on the effect of spinal PKC inhibition on pain behaviors after CFA with mixed results [20,34]. Nonetheless, PKCα or PKCγ are apparently involved in the biochemical modifications of NMDA receptors and/or GluR receptors translocation/internalisation within the lumbar spinal cord of the CFA model [34,35]. Importantly, a very recent study has shown that spinal PKMζ underlies the initiation but also the maintenance mechanism of persistent nociceptive sensitization [24]. In a model of "hyperalgesic priming", blockage of PKMζ at the time of intraplantar IL-6 inhibited allodynia. PKMζ inhibition before subsequent challenge with intraplantar PGE2 or intrathecal mGlu1/5 agonist, when IL-6-induced allodynia was completely resolved, also abolished pain behaviors. Thus, all these data suggest a specific role of PKCζ/PKMζ in pain and spinal persistent nociceptive processing, especially following peripheral inflammation. Indeed, intrathecal administration of ZIP did not modify neuropathy-induced mechanical hypersensitivity. Moreover, Li et al, found that spinal ZIP administration did not modify mechanical allodynia in neuropathic mice, there was no change in lumbar PKMζ/p-PKMζ expression and ZIP did not affect neuropathy-induced increased EPSCs from lamina II neurons [36]. However, they demonstrated a role of PKMζ in the anterior cingulated cortex in neuropathic pain hypersensitivity [36]. This differential involvement of spinal PKMζ is of crucial importance to understand the difference of the molecular mechanisms of persistent spinal nociceptive processing between inflammatory and neuropathic pain. It is plausible that the type of stimulus at the periphery and the release of specific algogenic substances following an inflammatory stimulus could specifically promote spinal PKMζ activation. For example, PKCζ has been reported to be involved in receptor signalling complexes such as TNF-α and IL1-β receptors and activation of NFkB transcription factor during immune reactions which can occur in inflammatory pain states. Further studies are definitely needed to clarify this differential involvement of PKCζ/PKMζ between inflammatory and neuropathic pain.

Next, we assessed the expression of PKCζ/PKMζ in naïve animals and whether the phosphorylated forms of PKCζ/PKMζ change in the ipsilateral lumbar dorsal horn after peripheral inflammation. In naïve animals, both mRNA isoforms coding specifically for PKCζ and PKMζ are present in the DRG and lumbar spinal cord. Using western immunoblotting, we confirmed expression of PKCζ protein in the lumbar spinal cord but also in the DRG and the hippocampus. However, the antidody used specifically recognized PKCζ but not PKMζ. Nonetheless, we found an expression of phospho-PKCζ/p-PKMζ mainly in small and medium DRG neurones of naïve animals. We therefore examined if p-PKCζ/p-PKMζ are expressed in central terminals of primary afferent fibres within the spinal cord of formalin rats. We did not observe any obvious co-staining of p-PKCζ/p-PKMζ with IB4 and CGRP in lumbar spinal cord. In addition, using lumbar rhizotomy, which causes a substantial loss of primary afferent terminals within the spinal cord [37,38], we still observed p-PKCζ/p-PKMζ staining in the dorsal horn, suggesting a predominantly intrinsic spinal source. Indeed, cells expressing p-PKCζ/p-PKMζ were neuronal as shown by co-expression with NeuN, a marker of neuronal nuclei and PKCζ is physically linked to NR2B-containing NMDA receptors using co-immunoprecipitation. These results suggest that PKCζ expression is restricted to intrinsic dorsal horn neurons within superficial laminae and could be an intracellular protein effector of the NMDA receptor complex.

We also conducted experiments to examine possible changes in the levels of phosphorylated (i.e. activated) PKCζ and PKMζ in both inflammatory models. We observed a significant increase of p-PKCζ and p-PKMζ in the ipsilateral superficial dorsal horn, predominantly in the medial part, compared to the contralateral side in both models treated either with saline, the scrambled peptide or ZIP using immunohistochemistry. Furthermore, we only detected a significant increase of p-PKMζ but not p-PKCζ expression in the ipsilateral dorsal horn compared to the contralateral side in formalin rats using western blot. Finally, ZIP did not affect the phosphorylation of PKMζ and PKCζ in both models, as previously demonstrated for PKMζ in hippocampal slices following electrical stimulation-induced LTP [18]. Therefore, the antinociceptive effect of this specific enzymatic site inhibitor may be due to a reduction of the phosphorylating capacity (i.e. catalytic activity) of mainly PKMζ, at least following formalin, on downstream targets rather than its ability to be phosphorylated and further activated.

In both inflammatory pain models, we found Fos upregulation in superficial and deep laminae of the dorsal horn, which was reduced by PKCζ/PKMζ inhibition, reinforcing the hypothesis of a profound effect on neuronal activity. This also suggests that PKCζ/PKMζ is an upstream regulator of Fos expression. PKCε also influences Fos expression in the lumbar dorsal horn following formalin, though its effects are mediated by inhibition of neurotransmitter release from primary afferent fibre terminals [25], while decreased expression of Fos in PKCγ knockout mice following formalin injection only occurs in laminae I-II [30]. Therefore, other PKCs, including PKCζ/PKMζ, are certainly involved in Fos expression and neuronal excitability, particularly in the deep dorsal horn. The deep dorsal horn neurons receive nociceptive inputs via interneurons and/or their dorsally extending dendrites. Thus, as PKCζ/PKMζ is primarily expressed in superficial dorsal horn, the involvement of this kinase on deep dorsal horn neurons activity is likely to be indirect. Interestingly, PI3K, mTOR, CAMKII, and ERK, expressed by dorsal horn neurons play a crucial role in spinal nociceptive plasticity, while also regulating the expression but probably not the phosphorylation of PKMζ in LTP [17-19,29,39-41]. Such effectors (e.g. CaMKII, PI3K...) may influence PKCζ/PKMζ activity, and ultimately contribute to spinal persistent nociceptive processing. We finally demonstrated a physical link between NR2B-containing NMDA receptors and PKCζ, therefore atypical PKCζ/PKMζ could participate in NMDA receptors activity in a context of chronic inflammatory pain but also through post-synaptic mGlu receptors [24]. Further studies are needed to investigate the upstream regulators (e.g. kinases) and downstream targets of spinal PKCζ/PKMζ in chronic inflammatory pain.

## Conclusions

In the present study, we have demonstrated that PKCζ especially PKMζ isoform may be a significant factor in spinal persistent nociceptive processing following peripheral inflammation. This role of PKCζ/PKMζ seems to be restricted to inflammatory pain since there was no involvement of this kinase in acute and neuropathic pain. Pharmacological targeting of PKCζ/PKMζ may be of therapeutic benefit in chronic inflammatory pain conditions.

## Materials and methods

### Animals

All experiments were carried out using adult male Wistar rats (220-250g, Harlan, UK or Central Biological Services, University College London, UK), housed in standard laboratory conditions with free access to food and water. Experiments were approved by the UK Home Office and followed guidelines set by the International Association for the Study for Pain [42].

### Behavioral testing

Experiments were performed in a quiet room by a single blinded experimenter using the method of equal blocks with randomization of treatments in order to avoid any uncontrollable environmental influence that might introduce variability in behavioral responses.

### Thermal and mechanical nociception tests in normal rats

Naïve rats (180-200g, Harlan, UK) were injected intrathecally with 10 μg of myristoylated PKCζ pseudosubstrate inhibitor, ZIP (Myr-SIYRRGARRWRKL-OH; Biosource International Inc., USA) previously used as a specific inhibitor of PKCζ/PKMζ in several studies [15,24,36,43] or myristoylated control scrambled peptide (Myr-RLYRKRIWRSAGR-OH; Genscript, USA). Thermal and mechanical thresholds were assessed 30, 60, 90 and 120 min post-injection using the thermal plantar test and automatic Von Frey test, respectively (for details see below).

### Assessment of locomotor function - Rotarod

Locomotor function and motor effects of drugs were assessed using an accelerating rotarod device (Bioseb, France). Naïve rats (180-200 g) underwent a training period over 2 consecutive days (1 trial/day) prior to testing of drug effects in order to ensure that they could remain on the rotarod at 4 revolutions per minute (rpm) for 5 min. On the day of drug administration, the apparatus was set to accelerate from 4 to 40 rpm over 5 min and the latency to fall was monitored. Locomotor function in the accelerating rotarod was assessed 30 and 60 min following intrathecal injection of ZIP or the scrambled peptide (both 10 μg/rat).

### Formalin test

Prior to being placed in a Plexiglas box for 30 min to acclimatize, rats received a direct intrathecal (i.t.) injection of 5 or 10 μg of myristoylated PKCζ pseudosubstrate inhibitor, ZIP, (Biosource International Inc., USA), control scrambled peptide (Genscript, USA) or 0.9% saline (*n *= 8 in each group). Thirty minutes later rats received 50 μl of 5% formalin injected subcutaneously (s.c.) into the plantar surface of the right hindpaw. Lifting, shaking, biting and licking of the injected paw were recorded by measuring the total duration of the response in seconds during the 60 min period following formalin administration. Data are presented in 5 min time bins. At the end of the test, animals were terminally anaesthetized and perfused (for details see below).

### Induction of mononeuropathy: chronic constriction nerve injury (CCI)

Unilateral peripheral mononeuropathy was induced according to the method previously described by [44]. Briefly, rats (150-175 g) were anesthetized with sodium pentobarbital (6%, 1 ml/kg, i.p.). After skin incision, the right sciatic nerve was exposed and 4 polyester sutures (MERSUTURE^® ^3-0, Ethicon, Johnson & Johnson, France) were tied loosely around at 1 mm intervals, so that the nerve was constricted but the circulation was not interrupted. The skin was then sutured (MONOCRYL^® ^5-0, Ethicon, Johnson & Johnson, Issy-les-Moulineaux, France). The animals were allowed to recover and were routinely monitored to assure good health. To examine mechanical allodynia (i.e. Von Frey test) and hyperalgesia (i.e. paw pressure test), we used two separate groups of animals. The right hindpaw was tested and measurements were taken on two separate days prior CCI surgery (baseline), 14 days after CCI induction (baseline post-CCI) and 30, 90 and 180 min or 30, 45, 120, 180 and 240 min for mechanical allodynia and hyperalgesia, respectively, after administration of a single direct intrathecal injection of ZIP or the scrambled peptide (both 10 μg/rat, *n *= 8 in each group).

### Induction of peripheral inflammation with CFA

Rats were injected with 50 μl of Complete Freund's Adjuvant (CFA; 1mg/ml *Mycobacterium Tuberculosis*; Sigma, UK) into the plantar surface of the right hindpaw to induce inflammation. The CFA-treated paw showed marked swelling within 2-3 hrs which persisted for several days. To examine mechanical and thermal hypersensitivity, the left and right hindpaws were tested alternatively and measurements were taken on two separate days prior to CFA administration, 24 hrs after CFA injection (baseline post-CFA) and 30, 90 and 180 min after administration of a single direct intrathecal injection of ZIP or the scrambled peptide (both 10 μg/rat, *n *= 8 in each group). At the end of the experiment, animals were terminally anaesthetized and perfused (for details see below).

### Thermal and Mechanical hypersensitivity

Each animal was placed in a clear acrylic cubicle (22 × 16.5 × 14 cm) on top of a glass floor in a temperature controlled room (~22°C) and allowed to acclimatize for 15 min before testing. Thermal responses were tested using the method described by [45]. Withdrawal latencies were averaged over three consecutive tests, at least 5 min apart, in response to the thermal challenge from a calibrated radiant light source (output of 190 mW/cm2). A cut-off of 20 s was imposed to prevent tissue damage.

Mechanical withdrawal thresholds were tested using a Dynamic Plantar Aesthesiometer (Ugo Basile, Italy). The stimulus was applied via an actuator filament (0.5 mm diameter) which, under computer control, applied a linearly increasing force ramp (2.5 g/sec, maximum force 50 g) to the plantar surface of the hindpaw. The force necessary to elicit paw withdrawal was recorded. The withdrawal threshold was calculated as the average of three consecutive tests, with at least 5 min between each test. A cut-off of 50 g which is the maximum force applied by the apparatus was used.

In order to assess mechanical hyperalgesia, a group of CCI rats were submitted to the paw pressure test previously described by [46]. Nociceptive thresholds, expressed in grams, were measured using an Ugo Basile analgesimeter (Bioseb, France) which applied an increasing pressure to the right hind paw until a squeak (vocalisation threshold) was elicited. The vocalisation threshold was measured 2 or 3 times before (baseline) and after ligatures (baseline post-CCI) in order to obtain two consecutive values that differed no more than 10%, and respecting an interval of at least 10 min between two measures. The maximal pressure (cut-off) applied was 750 g.

### Direct intrathecal injection via lumbar puncture

Intrathecal (i.t.) injections were performed under isoflurane anaesthesia (4% induction, 2% maintenance) according to the method previously described [47]. The anaesthetised rat was held firmly at the pelvic girdle and drug was delivered (10 μl/rat) using a 25-gauge × 1-inch needle connected to a 25 μl Hamilton syringe inserted into the subarachnoidal space between lumbar vertebrae L5 and L6, eliciting a tail flick. The syringe was held in position for a few seconds after the injection.

### In vivo Electrophysiology - Formalin Test

#### Preparation

Anaesthesia of rats was induced using 4-5% isoflurane (66% N_2_O & 33% O_2_) and a tracheal cannula was inserted, once areflexic. Rats were placed in a stereotaxic frame to ensure stability during electrophysiological recordings, and core body temperature was maintained at 36.5-37°C using a heating blanket connected to a thermal rectal probe. Anaesthesia was reduced to 2.5% isoflurane, and a laminectomy was performed at the L1-L3 vertebral level, exposing the L4-L5 segments of the spinal cord. Anaesthesia was then reduced to 1.5% isoflurane and was maintained at this level for the duration of the experiment.

Identification of different spinal cell types was achieved by tapping at the hindpaw receptive field. Extracellular recordings from single convergent deep dorsal horn (> 600 μm) wide dynamic range neurons (WDRs) were made using parylene coated tungsten electrodes (A-M Systems, Washington USA). Cells were characterised prior to formalin administration. First, cells were stimulated electrically. A train of 16 transcutaneous electrical stimuli (2 ms wide pulses, 0.5 Hz) was applied at three times the threshold current for C-fibres via two stimulating needles inserted under the skin of the hindpaw, in order to assess primary afferent fibre input. Next, the receptive field was stimulated thermally by applying a constant jet of water using a needle and syringe. Both an innocuous (35°C) and noxious (48°C) temperature was applied in order to indicate a strong C-fibre input to the WDR neuron being recorded, which has been shown to be required for the response to subcutaneous formalin [48].

#### Administration of Drug and Formalin

Following cell characterization, 10 μg of the myristoylated PKCζ pseudosubstrate inhibitor, ZIP (*n *= 8), in a total volume of 50 μl, was applied directly onto the surface of the spinal cord using a Hamilton syringe, 30 min prior to the injection of formalin. A separate group of control animals (*n *= 8) received the control scrambled peptide (10 μg/50 μl).

Formalin (5%, 50 μl) was injected subcutaneously into the centre of the hindpaw receptive field and the firing response of the WDR neuron was followed for the subsequent 70 min. Activity was displayed as a rate recording and quantified in 10 min time bins. Data was captured and analyzed by a CED 1401 interface coupled to a Pentium computer with Spike 2 software (Cambridge Electronic Design, UK; rate function) and presented as number of action potentials (APs).

### Lumbar rhizotomy surgery

Lumbar rhizotomy was carried out as previously described by [37] and [38]. In brief, animals were deeply anaesthetized by intraperitoneal (i.p.) injection of a mixture of medetomidine (0.25 mg/kg) and ketamine (60 mg/kg). Skin and muscle incisions were made to expose the vertebral laminae. The intervertebral foraminae were enlarged and L4, L5 and L6 dorsal roots were exposed and crushed for 10s each. The muscle and skin were then closed with 4.0 sutures. Seven days after surgery, animals were deeply anaesthetised and perfused (see below). Tissue processing (lumbar spinal cord) and immunostaining were conducted as described below.

### Immunohistochemistry for phospho-PKCζ and Fos

At the end of the formalin and CFA behavioral experiments, animals were terminally anaesthetised using pentobarbitone and quickly perfused transcardially with saline followed by 4% paraformaldehyde (PFA) with 15% of a saturated solution of picric acid to reduce substantial dephosphorylation. After perfusion, the lumbar spinal cord was excised, post-fixed for 4 hrs in the perfusion fixative, cryoprotected in 20% sucrose in 0.1 M phosphate buffer (PB) overnight at 4°C, and then frozen in O.C.T compound. Transverse sections (30 μm) were cut on a cryostat and thaw-mounted onto glass slides. Sections were stained for p-PKCζ immunohistochemistry as follows: after 3 washes in PBS, sections were incubated for 48 hrs at 4°C with a rabbit primary antibody for anti-phospho-PKCζ (anti-p-PKCζ, Thr 410 sc-12894-R; 1:100; Santa Cruz Biotechnology, USA) followed by a secondary antibody solution for 4 hrs (goat anti-rabbit IgG-conjugated Alexa Fluor 488™; 1:1000; Molecular Probes, USA), as previously described [22]. Slides were washed in PBS and cover-slipped with Vectashield mounting medium (Vector Laboratories, CA, USA).

Sections were visualized under a Zeiss Axioplan 2 fluorescent microscope running Axiovision 3.1 image analysis software and classified according to spinal level (L4, L5 and L6). Quantitative assessment of p-PKCζ/p-PKMζ staining was carried out by determining the immunofluorescence intensity using grey scale within a fixed area of the dorsal horn of the spinal cord (ipsilateral and contralateral to injury), as described by [19]. A box measuring 10^4 ^μm^2 ^(100 × 100) was placed over the medial part of the superficial dorsal horn and the mean intensity of each area recorded. The measurement protocol was carried out on L4-L6 spinal sections of each animal (at least 5 sections per animal). The background fluorescence intensity of each tissue section was also determined and subtracted from recorded values. Data are presented as mean ± s.e.m. of immunofluorescence intensity (arbitrary unit) of p-PKCζ/p-PKMζ staining in ipsilateral and contralateral dorsal horn for each group.

To determine the cellular distribution of p-PKCζ/p-PKMζ, sections from formalin rats were counterstained with primary antibodies against markers for neurons [mouse anti-neuronal nuclei (anti-NeuN); 1:500; Chemicon, Hampshire, UK], astrocytes [mouse anti-glial fibrillary acidic protein (anti-GFAP); 1:1000; Abcam, Cambridge, UK], microglial cells [rabbit anti-ionized calcium binding adaptor molecule 1 (anti-Iba1); 1:100; Wako Pure Chemical Industries Ltd, Japan], peptidergic primary afferent fibres [sheep anti-calcitonin gene related peptide (anti-CGRP); 1:800; Biomol International LP, USA], and non-peptidergic primary afferent fibres, [biotinylated lectin IB4 (anti-IB4); 1:1000; Sigma, UK], followed by the appropriate secondary antibody solution [goat anti-rabbit IgG-conjugated Alexa Fluor 546™; (1:1000; Molecular Probes, USA) for NeuN, GFAP and Iba1 or anti-sheep Cy3 (1:400; Stratech Scientific Ltd, UK) and extra avidin-TRICT [1:200; Sigma, UK; for CGRP and IB4].

Double immunostaining for p-PKCζ/p-PKMζ with CGRP or IB4 (see protocol above for CGRP or IB4 staining), was also conducted on lumbar spinal cord sections from a group of animals which had rhizotomy surgery (see 2.5). Following CGRP or IB4 staining, sections were incubated with rabbit anti-p-PKCζ (Thr 410, sc-12894-R; 1:100; Santa Cruz Biotechnology, USA) followed by secondary antibody solution (goat anti-rabbit IgG-conjugated Alexa Fluor 488™; 1:1000; Molecular Probes, USA). Slides were washed in PBS, cover-slipped and visualised under a Zeiss Axioplan 2 Microscope (Zeiss, Hertfordshire, UK).

The specificity of p-PKCζ/p-PKMζ immunostaining was verified by preabsorption of the primary antibody (Thr 410, sc-12894-R; 1:100; Santa Cruz Biotechnology, USA) with a 5 times excess (weight/weight) of the peptide used to generate the antibody (sc-12891 P, blocking peptide) overnight at 4°C. The pre-absorbed peptide was then centrifuged 20 min at 10 000 rpm and the top half of the liquid was collected and applied on tissue sections. A positive control (antibody alone at the same dilution) and a negative control (lack of primary antibody) were run in parallel and applied on sister sections. The antibody against IB4 was added to all tubes include in this specificity test. Immunostaining was revealed by incubation with secondary antibody (goat anti-rabbit IgG-conjugated Alexa Fluor 546™; 1:1000; Molecular Probes, USA) for 2 hours.

Sections were processed for Fos staining as follows: following 3 washes in PBS, sections were incubated overnight, at room temperature with the primary rabbit anti-Fos antiserum (1:2500 in PBST-Azide, AB5; Oncogene Science, Uniondale, NY, USA). Next, sections were incubated for 4 hrs with secondary antibody (AlexaFluor™ 488 goat anti-rabbit IgG; 1:1000; Molecular Probes, USA). Slides were washed in PBS and cover-slipped with Vectashield mounting medium (Vector Laboratories, CA, USA). From each animal 4-6 sections (L4-L6) were randomly selected for counting Fos positive cells by a blinded investigator and an average of these counts was taken.

### Western immunoblotting and immunoprecipitation

Naïve rats anaesthetized with urethane were sacrificed by decapitation and fresh dorsal roots ganglia (DRG), spinal cord (dorsal horn only) and hippocampus were dissected out and snap frozen. Tissue samples were subsequently homogenized in RIPA (Radioimmunoprecipitation assay) buffer (50 mM Tris HCl pH 7.5, 150 mM NaCl, 1 mM EDTA, 1% NP-40, 0.1% SDS) + 0.5% DOC (Deoxycholic acid) + Complete protease inhibitor cocktail) using a glass homogenizer. Homogenate was then centrifuged at 14000 rpm for 10 min at 4°C and supernatant was collected. DRG, spinal dorsal horn and hippocampus whole cell lysates were next titrated to determine their protein concentrations using a BCA Protein Assay kit (Pierce, UK).

In a separate experiment, ipsilateral and contralateral sides of superficial spinal dorsal horn from scrambled peptide-treated animals (n *= *4) and ZIP-treated animals (n *= *4) were dissected out 60 min after intraplantar formalin 5%. Following the dissection, tissues samples followed the same procedure as above.

For western immunoblotting, lamaelli loading buffer was added to DRG, dorsal horn and hippocampus protein lysates (30 μg) from naïve animals and samples were incubated at 70°C for 30 min. Samples were then loaded onto 8% gels and separated by sodium dodecyl sulphate polyacrylamide gel electrophoresis (SDS-PAGE). After protein transfer to nitrocellulose membranes, membranes were incubated with rabbit anti-PKCζ primary antibody (1:500, ab59364, Abcam, UK) and rabbit anti-neuronal ßIII-tubulin (1:3000, ab18207, Abcam, UK), which served as a loading control, overnight at 4°C, in order to identify PKCζ/PKMζ expression in either DRG, lumbar spinal cord or hippocampus in naïve rats.

Samples from ipsilateral and contralateral sides of superficial spinal dorsal horn from formalin rats were submitted to the same procedure as above and finally transfer to nitrocellulose membranes. Membranes were then incubated with rabbit anti-pPKCζ primary antibody (Thr 410 sc-12894-R; 1:500; Santa Cruz Biotechnology, USA) overnight at 4°C, in order to quantify p-PKCζ and p-PKMζ expression between ipsilateral and contralateral dorsal horn of formalin animals.

Following incubation with dye-linked donkey anti-rabbit IR800 or goat anti-mouse IR600 secondary antibody, proteins were revealed using the Odyssey fluorescence detection system (Licor, UK). For p-PKCζ and p-PKMζ expression levels, bands were quantified by densitometric analysis using Image J software. Results are expressed as mean ± s.e.m. of the densitometric analysis (arbitrary unit) of the phosphorylation of p-PKCζ and p-PKMζ expression levels in the ipsilateral and contralateral dorsal horn for each group (both ipsilateral and contralateral samples were run on the same gel).

For immunoprecipitation of NR2B subunits, Dynabeads^® ^Protein G (Invitrogen Ltd, UK) were washed and coupled with 5 μg of rabbit anti-NR2B antibody (06-600, Upstate, USA). Next 500 μg of spinal dorsal horn lysate from naïve animals was added to the antibody-dynabeads complex and allowed to incubate overnight for capture of target antigen. Captured protein was eluted from the dynabeads by resuspension in 40 μl of Lamaelli loading buffer and heating at 70°C for 30 min. Samples were then separated onto 8% gels by SDS-PAGE. Normal spinal lysates samples were run alongside IP samples as positive controls. Following electrophoresis and protein transfer to nitrocellulose membranes, membranes were incubated with primary antibody overnight at 4°C. Antibodies used were mouse anti-NR2B (1:500, 75-101, Neuromab, USA), rabbit anti-PKCζ (1:500, ab59364, Abcam, UK). Membranes were also probed with rabbit anti-CREB (1:500, ab5803, Abcam, UK) and rabbit anti-P_2_X_3 _(1:500, ab10269, Abcam, UK) antibodies used as negative controls. Following incubation with dye-linked donkey anti-rabbit IR800 or goat anti-mouse IR600 secondary antibody, proteins were revealed using the Odyssey fluorescence detection system (Licor, UK).

### RT-PCR

Total RNA from cortex, hippocampus, spinal cord and DRG from naïve rats was used to synthesize cDNA with the SuperScript™ II Reverserve Transcriptase kit (Invitrogen). 1 μg of cDNA was used for PCR (50 μl final reaction volume) and amplified according to [11]. Cycle parameters were set as 34 cycles at 94°C for 30 s, 60°C for 1 min and 72°C for 1 min, with a final step of 72°C for 10 min. For amplification, F 5'-CCATGCCCAGCAGGACCACC-3' and 5'-CCTTCTATTAGATGCCTGCTCTCC-3' were used as specific forward primers for PKCζ and PKMζ, respectively and R 5'-TGAAGGAAGGTCTACACCATCGTTC-3' was the reverse primer for both.

### Statistical analysis

All data are presented as mean ± s.e.m. Cell characteristics from PKCζ/PKMζ pseudosubstrate inhibitor or scrambled peptide groups were compared by one-way analysis of variance (ANOVA), followed by Bonferroni's post-test. Formalin neuronal and behavioral response time-course data for formalin and CFA rats were compared between treatment groups by two-way repeated measures (RM) ANOVA, followed by Bonferroni's post-test. Total activity in the first and second phases was compared between treatment groups by one-way ANOVA, followed by Bonferroni's post-test. Immunostaining and western immunoblotting of p-PKCζ/p-PKMζ and Fos was analyzed by one-way and two-way ANOVA, respectively, followed by Bonferroni post-tests. Statistical analyses were carried out using GraphPad Prism v.4 software (GraphPad Software Inc., San Diego, CA, USA).

## Competing interests

The authors declare that they have no competing interests.

## Authors' contributions

FM and RM overall experimental design, collected the behavioral data, performed RT-PCR, participated to immunohistochemistry and western blotting experiments. RM performed the electrophysiology experiment. PY realised lumbar rhizotomy surgery and contributed to RT-PCR. MC, EM and SP performed immunohistochemitry and western blotting experiments. FM, RD and SP performed analysis and drafted the manuscript. AH and SB overall supervision of the experiments and writing of the manuscript. All authors read and approved the final manuscript.
